# Interactive Multi‐Stage Robotic Positioner for Intra‐Operative MRI‐Guided Stereotactic Neurosurgery

**DOI:** 10.1002/advs.202305495

**Published:** 2023-12-10

**Authors:** Zhuoliang He, Jing Dai, Justin Di‐Lang Ho, Hon‐Sing Tong, Xiaomei Wang, Ge Fang, Liyuan Liang, Chim‐Lee Cheung, Ziyan Guo, Hing‐Chiu Chang, Iulian Iordachita, Russell H. Taylor, Wai‐Sang Poon, Danny Tat‐Ming Chan, Ka‐Wai Kwok

**Affiliations:** ^1^ Department of Mechanical Engineering The University of Hong Kong Hong Kong 999077 China; ^2^ Department of Biomedical Engineering The Chinese University of Hong Kong Hong Kong 999077 China; ^3^ Department of Medical Physics and Biomedical Engineering University College London London WC1E 6BT UK; ^4^ Department of Mechanical Engineering and Laboratory for Computational Sensing and Robotics Johns Hopkins University Baltimore MD 21218 USA; ^5^ Department of Computer Science and Laboratory for Computational Sensing and Robotics Johns Hopkins University Baltimore MD 21218 USA; ^6^ Division of Neurosurgery Department of Surgery Prince of Wales Hospital The Chinese University of Hong Kong Hong Kong 999077 China; ^7^ Multi‐Scale Medical Robotics Center Hong Kong 999077 China; ^8^ Neuromedicine Center Shenzhen Hospital, The University of Hong Kong Shenzhen 518053 China; ^9^ Wellcome/EPSRC Centre for Interventional and Surgical Sciences University College London London WC1E 6BT UK

**Keywords:** frameless stereotaxis, MRI‐guided interventions, soft robotics, stereotactic neurosurgery, surgical robotics

## Abstract

Magnetic resonance imaging (MRI) demonstrates clear advantages over other imaging modalities in neurosurgery with its ability to delineate critical neurovascular structures and cancerous tissue in high‐resolution 3D anatomical roadmaps. However, its application has been limited to interventions performed based on static pre/post‐operative imaging, where errors accrue from stereotactic frame setup, image registration, and brain shift. To leverage the powerful intra‐operative functions of MRI, e.g., instrument tracking, monitoring of physiological changes and tissue temperature in MRI‐guided bilateral stereotactic neurosurgery, a multi‐stage robotic positioner is proposed. The system positions cannula/needle instruments using a lightweight (203 g) and compact (Ø97 × 81 mm) skull‐mounted structure that fits within most standard imaging head coils. With optimized design in soft robotics, the system operates in two stages: i) manual coarse adjustment performed interactively by the surgeon (workspace of ±30°), ii) automatic fine adjustment with precise (<0.2° orientation error), responsive (1.4 Hz bandwidth), and high‐resolution (0.058°) soft robotic positioning. Orientation locking provides sufficient transmission stiffness (4.07 N/mm) for instrument advancement. The system's clinical workflow and accuracy is validated with lab‐based (<0.8 mm) and MRI‐based testing on skull phantoms (<1.7 mm) and a cadaver subject (<2.2 mm). Custom‐made wireless omni‐directional tracking markers facilitated robot registration under MRI.

## Graphical abstract

To leverage the intra‐operative functions of MRI, e.g., instrument tracking, monitoring of physiological changes and tissue temperature in MRI‐guided stereotactic neurosurgery, a robotic positioner is proposed in this study. The compact and skull‐mounted robot incorporates soft robotics and operates in manual and automatic stages. The system is validated by a cadaver trial. Custom‐made wireless MRI tracking markers are also discussed.

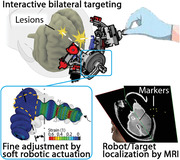

## 1 Introduction

**Figure 1:**
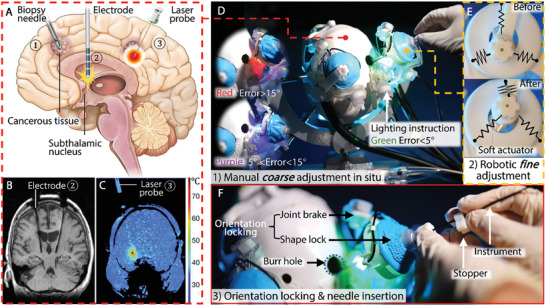
Clinical background and workflow of the proposed robotic system. A) Stereotactic approaches for ①biopsy, ②deep brain stimulation (DBS), ③ablation. B) Coronal MRI showing lead placement in a patient undergoing DBS surgery; C) MR thermometry of a localized ablation for mesial temporal lobe epilepsy. relative temperature change from baseline following ablation exceeded 30 °C. D) Manual *coarse* adjustment of the instrument guide. Interactive lighting instruction is incorporated during hands on adjustment to indicate errors >15° (red), between 5° and 15° (purple), and <5° (green). E) Robotic *fine* adjustment of the instrument guide conducted by soft actuators. F) Instrument (cannula) insertion with robot locked. The insertion depth can be measured and pre‐set manually using a stopper. Image source:^[^ ^23^, ^24^ ^]^.

Magnetic resonance (MR) imaging (MRI) plays a critical role in the management of debilitating diseases including heart rhythm disorder, neurological abnormalities, and many forms of cancer. Providing high‐contrast 2D and 3D images of soft tissue without harmful ionizing radiation, it is a compelling and powerful tool for clinicians. In the realm of neurosurgery, MRI demonstrates clear advantages over other conventional imaging modalities (e.g., computed tomography (CT) and ultrasound) with its ability to precisely delineate critical neurovascular structures and cancerous tissue, allowing the formation of high‐resolution 3D anatomical roadmaps for guiding pre‐operative (pre‐op) planning and post‐operative (post‐op) monitoring. The treatment of many neurological diseases such as brain tumors, Parkinson's disease, deep depression, and Alzheimer's involves the precise insertion of instruments, such as biopsy needles (**Figure** **1**A), electrodes (Figure 1B), or thermal therapy laser probes (Figure 1C) toward target structures (e.g., subthalamic nucleus (STN)). Traditionally, this process relies on a large frame (i.e., stereotactic frame) that is rigidly mounted to the skull to allow adjustment of the instrument orientation with respect to a 3D coordinate system. The instrument insertion process is conventionally guided only by pre‐op MRI and CT images registered to the stereotactic frame, with the exception of deep brain stimulation (DBS) where the brain electrical activity is monitored by microelectrode recording^[^ ^1^ ^]^ during insertion. A major limitation of current practice is that procedural planning and intervention are performed based on static pre‐op and post‐op imaging. However, non‐trivial amounts of error can accrue from sources such as the intrinsic mechanical error in the stereotactic frame, image registration error and differences in patient positioning in pre‐op imaging versus surgery. A phenomenon known as brain shift can also displace the brain and target anatomy up to 10–30 mm when creating the instrument entry site (or burr hole).^[^ ^2^ ^]^

Despite MRI being positioned as a key part in conventional neurosurgery, its application has largely been limited to pre‐op planning and post‐op management. However, recent advances in MRI hardware, imaging protocols, and position tracking coils have led to its emergence as an effective choice for interventions under intra‐operative (intra‐op) guidance.^[^ ^3^ ^]^ Specifically, MRI can be leveraged to measure tissue temperature (i.e., MR thermometry), perform in‐situ instrument tracking, and monitor physiological changes in tissue, all of which are critical to many neurosurgical procedures. Several commercial products have aimed to build on conventional stereotactic approaches by developing MR‐compatible frames, with notable examples such as the *ClearPoint* system,^[^ ^4^ ^]^ the *Nexframe* system by Medtronic,^[^ ^5^ ^]^ and the *AXiiiS* stereotactic frame by Monteris.^[^ ^6^ ^]^ These systems allow the performing of instrument positioning and insertion within the MRI scanner.^[^ ^7^ ^]^ However, notably, these systems are solely manual driven, requiring the surgeon to control the entire instrument positioning process. For the *Nexframe* and *AXiiiS* frames, manual driving can severely disrupt the surgical workflow because the patient must be moved in and out of the MRI isocenter to make any trajectory adjustments. This can require repeated rounds of imaging and adjustment before adequate targeting precision is achieved, i.e., <3 mm error.^[^ ^1^ ^]^ The *Clearpoint* system partially relieves this by providing a remote manual controller for the surgeon such that the patient can remain inside the MRI bore, however, manually‐intensive adjustments are still required throughout the procedure. Multiple readjustments must be made through the manipulation of numerous independent control knobs connected via long flexible transmissions, without intuitive feedback.^[^ ^4^ ^]^

These challenges have sparked the development of MR‐compatible surgical robots which can smoothly integrate MRI's intra‐op capabilities with minimal hindrance to the surgical workflow. With the field of neurosurgery being an early adopter of robotic assistance, the trend has remained true for MR‐compatible robots, with the first robotic stereotactic system being presented by Masamune et al.^[^ ^8^ ^]^ in 1995. Since then, the development of MRI‐guided robotics has spread across applications including breast biopsy,^[^ ^9^ ^]^ percutaneous liver ablation,^[^ ^10^ ^]^ transrectal prostate biopsy,^[^ ^11^ ^]^ and transoral laser ablation.^[^ ^12^ ^]^ After Masamune, researchers have developed MRI‐compatible neurosurgery robots with a wide variety of mechanical designs, formfactors, and functionality. Some used alternative actuation approaches such as pneumatics,^[^ ^13^ ^]^ tendon‐driven transmissions,^[^ ^14^ ^]^ or piezoelectric motors.^[^ ^15^ ^]^ For example, Stoianovici et al.^[^ ^16^, ^17^ ^]^ presented a needle positioning robot with custom‐made MR‐safe pneumatic motors for control over a remote‐center‐of‐motion (RCM) mechanism with three additional manual degrees of freedom (DoFs) that must be locked independently by the surgeon. The robot could be mounted to the patient table or to a Mayfield head frame, although its size would also only accommodate unilateral procedures. With the aim to develop a needle guidance robot for bilateral DBS, Guo et al.^[^ ^18^ ^]^ presented a relatively compact and lightweight pair of robots that could operate within a conventional cage‐style head coil. The design implemented fully robotic actuation achieved in part by their hybrid actuation mechanism which employed long‐range hydraulics (≈10 m) that converted to a tendon‐driven parallel five‐bar mechanism. Although the skull‐mounted unit of the robot was compact, the actuation conversion involves a large actuation module tethered to the patient skull. Additionally, transmission losses manifesting as backlash were present due to the use of slender bar linkages combined with the high elasticity of MR‐safe/conditional tendons. As it stands, few robotic commercial products and research‐based prototypes have been developed for MRI‐based instrument positioning, particularly those that are small enough to utilize conventional imaging head coils and further, allow for bilateral procedures. FDA‐cleared examples include the *NeuroBlate* system by Monteris Medical^[^ ^19^, ^20^ ^]^ and *Exablate Neuro* system by Insightec^[^ ^21^ ^]^ which leverage MRI's real‐time thermometry for treatment monitoring, but are only compatible with their proprietary instruments. A fully robotic system under development by AiM Medical Robotics uses a semi‐circular gantry design, which is not compatible with conventional head coils and for bilateral procedures.

Across the systems developed, a notable compromise can be seen between workspace coverage, level of automation, and overall formfactor/size: to achieve fully robotic targeting over a sufficient surgical workspace, large ancillary actuation units or supporting frames are often needed. Alternatively, other approaches may opt for only robotizing several key DoFs (e.g., instrument insertion and rolling) while relying on basic manually‐adjusted frames to position the overall robot, which can hinder a smooth surgical workflow and ease of use. Considering these challenges and trade‐offs, we have developed an MRI‐compatible instrument positioner^[^ ^22^ ^]^ which leverages soft robotics in an intuitive multi‐stage methodology. Although typically associated with compliant and inexact motion, we utilize soft robotics for its miniaturization potential, and uniquely optimize its design with reinforcements to ensure highly precise motions. As a result, the robotic system is compact enough to perform bilateral procedures inside conventional head coils. Overall robot operation begins with the surgeon manually adjusting the instrument orientation with interactive lighting alerts, followed by automated soft robotic fine adjustment toward the target. Intuitive hands‐on operation is achieved by centering the robot design on a concentric five‐bar linkage structure that maintains a RCM about the targeted burr hole. Even in sacrificing fully automated instrument positioning for a smaller and lightweight formfactor, we still fulfill key needs of cannula/needle‐based instrument neurosurgical procedures including sufficient workspace, clear burr hole visibility, robust orientation locking, and intuitive operation. The key contributions of our work are summarised below:

1) Development of a multi‐stage robotic positioner for MRI‐guided stereotactic neurosurgery which incorporates intuitive, hands‐on initial adjustment, followed by soft‐robotic final positioning. While maintaining a sufficient workspace (±35°), the positioner remains lightweight (203 g) and compact (Ø97 mm × 81 mm height), enabling skull‐mounted usage within most standard imaging head coils and in a bilateral configuration.
2) Formation of a precise soft robotic instrument positioning stage (<0.2° orientation error) via finite element analysis (FEA)‐based design and optimization of the fluid‐driven soft actuator architecture, paired with robust orientation locking across both stages. Experimental validation was carried out to evaluate overall targeting error (<1 mm), transmission stiffness, frequency response, and durability.
3) MRI‐based validation of overall system workflow, including robot registration with custom‐made omni‐directional MRI markers, and targeting accuracy testing in phantoms and a cadaver subject. Real‐time positional feedback is provided by optical encoders throughout the procedure under MRI. No notable reduction in imaging quality was observed during operation.

## 2 Results

The proposed robotic platform is designed to assist the surgeon in performing intra‐op MRI‐guided stereotactic neurosurgeries, in particular those cannula/needle targeting involved interventions, such as biopsy, injection, ablation, catheter placement, stereo electroencephalography (sEEG) and deep brain stimulat (DBS). For intra‐op MRI‐guided DBS, key targets are the STN and internal globus pallidus (GPi). These are situated in the deep region of the brain (average 90.4 mm^[^ ^25^ ^]^ beneath the skull) and demands electrode placement error of less than 3 mm^[^ ^1^ ^]^ to maintain adequate stimulation effect. The design of robot aims to fulfill several key criteria necessary for performing stereotactic instrument positioning under MRI. To leverage the imaging capabilities of MRI for neurosurgery, the robot should be able to fit and operate within the compact space of an imaging head coil. Although conventional helmet‐like head coils are often preferred by radiologists for their superior imaging quality, their closed design severely inhibits access to the frontal and parietal skull. Therefore, our robotic system is compatible with split or birdcage head coils, which provide a degree of access to the patient's skull while still having greater imaging quality than body or loop coils. The robotic platform itself consists of two independent instrument‐positioning robots which can be used to perform both unilateral and bilateral procedures as shown in Figure 1D. In particular, for certain sEEG applications where over 10 electrodes will be inserted, halving the operational time could significantly smoothen the workflow. The robots are lightweight (203 g each) and compact (Ø97 mm × 81 mm height each) allowing them to be mounted simultaneously to the skull with reduced patient discomfort. Each robot can be attached to the patient via the use of small, independent mount, which lend themselves to high flexibility when positioning each robot, particularly in bilateral configurations. As compared to table‐mounted robots, the skull‐mounted approach generally allows greater security in the case of undesired patient movement, due to rigid fixation to the patient.

The proposed robot system aims to strike a balance between workspace, automation, and compactness through a semi‐automated design that operates in two stages: 1) manual coarse adjustment within a large workspace (±30°) performed interactively by the surgeon, as in Figure 1D, followed by 2) automated fine adjustment within a localized motion range (±5°) performed by soft fluid‐driven actuators, as in Figure 1E and Movie S1 (Supporting Information). The major portion of instrument positioning is designated for the surgeon's hands‐on interaction with the system, which greatly reduces the challenges of integrating wide‐range actuation mechanisms and can simplify the overall mechanical system design. The surgeon can freely move the instrument guide in two DoFs (pitch and yaw) about the RCM during the coarse adjustment stage within a workspace sufficient (pitch angle of ±33° and yaw angle of ±26°^[^ ^18^ ^]^) for most neurosurgical procedures. The support of fiber optic lighting built into the robot base provides signals (Figure 1D) to the surgeon during *coarse* adjustment to indicate that the angular discrepancy is within 5° from the planned trajectory. After which, the *coarse* adjustment mechanism is locked into position for further robotic *fine* adjustment. The soft robot‐driven delta mechanism (Figure 1E) coupled to the instrument guide provides the final adjustment within ±5°, paired with another dedicated orientational shape locking (Figure 1F). The clinical workflow with the proposed robotic system is summarized in Movie S1 (Supporting Information), comprising three main steps as follows:

Step 1 – Planning: at the day of operation, pre‐op MR images are taken for planning the positioning of the skull burr hole/s according to the location of target anatomy. Subsequently, the robot mounting point/s will be determined to ensure that the target anatomy is within the robot workspace, while the burr hole remains adequately visible to the surgeon. After robot mounting, the patient is moved into the MR scanner isocenter for registration and re‐acquisition of the target anatomy location.
Step 2 – Instrument orientation adjustment: once registration is complete, the patient is moved out of the isocenter for the surgeon's access to the patient and robot. Targeting is then performed in two steps, starting with manual coarse adjustment. The surgeon first grasps the robot instrument guide and orients it approximately toward the direction of the planned trajectory based on pre‐op images. In‐built fiber‐optic lighting indicates to the surgeon their angulation error w.r.t. the planned trajectory, with different colors assigned to errors > 20° (red), between 5° and 20° (purple), and < 5° (green), as in Figure 1D. Once the surgeon has oriented the instrument guide with error < 5°, the coarse adjustment mechanism is remotely locked. The automated fine adjustment stage then robotically further positions the instrument guide to the planned trajectory (Figure 1E), followed by granular jamming to lock its orientation.
Stage 3 – Intervention: the insertion depth of the instrument (e.g., a DBS cannula) is set with the assistance of a stopper (Figure 1F). The surgeon then manually inserts the instrument through the robot instrument guide (Figure 1F). Lastly, the patient is then returned to the MRI isocenter for verification that the instrument targeting was successful and within expected tolerances, i.e., < 3 mm error for DBS. Subsequent real‐time MRI can be performed to monitor the interventional procedures, e.g., tumor ablation.

### 2.1 Interactive Five‐Bar Mechanism

**Figure 2:**
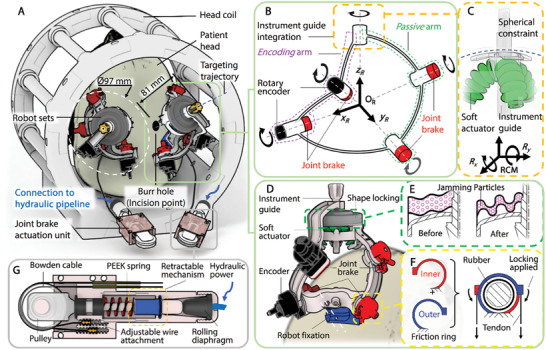
Overview of the robotic system implementation within an MRI head coil. A) Conceptual illustration of two robots mounted on the patient head within a head coil for bilateral procedures. Each robot is mounted above a respective burr hole (incision point) while allowing sufficient visibility and access for the surgeon. B) Schematic diagram highlighting the robot's *encoding* and *passive* arms of the five‐bar linkage mechanism. Both arms are integrated with joint brakes, whereas the *encoding* arm also includes rotary encoders. C) Three soft actuators pushing against the instrument guide following the robot RCM. D) Rendered CAD/CAM model showing the robot design. Compact fixation is designed to mount the robots onto the skull independently. MR‐safe encoders are integrated to provide closed‐loop feedback. Instrument guide orientation locking is achieved through granular jamming and tendon‐driven braking. E) Cross section view of the granular jamming module, showing its shape‐locking effect after inducing a vacuum. F) Schematic diagram of the tendon‐driven braking mechanism integrated into the robot joints. G) Cross section view of the joint brake actuation unit, which is placed ≈200 mm away from the robot and head coil.

To allow preservation of visibility and access to the surgical field for managing operative risks, e.g., bleeding, hematoma or/and embolic events, the positioner's instrument guide is designed to be elevated (35–40 mm from the bottom surface) above the burr hole, creating an exposure clearance. The instrument guide is therefore an overhang structure extended from the positioner base, which is anchored away from the burr hole. However, this cantilever‐type design inevitably creates a moment arm to support the overhanging components. As compared to existing manual approaches, e.g., Nexframe and Clearpoint, which obstruct the burr hole for direct structural support, maintaining architecture rigidity can be challenging. To this end, a positioner with a 5‐bar spherical linkage design (**Figure** **2**A,B) is proposed, featuring a reinforced RCM spherical constraint. Its mechanical architecture can be seen as a two‐arm parallel mechanism. The two serial two‐linkage spherical arms extend from the base linkage of the positioner, namely the *encoding arm* and *passive arm*, intersected at their distal joints to form a closed 5‐bar chain, as in Figure 2B. It shares essential advantages over single serial‐linkage arms, presenting higher positioning accuracy, lower mass/inertia properties, and greater structural rigidity (i.e., stiffness‐to‐mass ratio).^[^ ^26^ ^]^ These features are inherent to their specific kinematic structure, which resists error accumulation in kinematic chains. Located at the intersection of two arms, the instrument guide, as an end effector, can be manipulated manually and robotically by coarse/fine adjustment mechanisms (Figure 2C–E). For the coarse manual adjustment, the operator handhelds the instrument guide to initiate a motion along its spherical RCM, as in Figure 2B. Loadings introduced by motions deviated from the working RCM can be effectively countered by the closed 5‐bar chain, which is reinforced by the two connected parallel linkages for a smooth and even manipulation along the working range. While parallel mechanisms are usually characterized by high stiffness, they are also subject to elastic deformation of the linkages, and assembly tolerances. With enough misalignment between joint axes, the intended RCM constraint would be voided. To solve this, the robot linkages were reinforced with stiffening ribs (Figure 2D), forming a “H” shape cross sectional design, while keeping thin (2 mm) for lightness. Ceramic bearings (MR series, NSK Ltd.) and high‐performance thermoplastic (i.e., PEEK) screw connections were also utilized as a support to the revolute joints to reduce assembly backlash. During manipulation, the orientation of the end‐effector, i.e., instrument guide, w.r.t the robot frame {*x_R_*,*y_R_*,*z_R_*} (Figure 2B) is translated into the positional feedbacks^[^ ^27^ ^]^ from optical encoders (refer to section Targeting kinematics under Experimental Section) installed on the two proximal joints of the *encoding arm*, which is coupled to the instrument guide. This allows constant orientational real‐time tracking for instrument positioning between MRI scans.

As the orientation of the instrument guide is coupled to the *passive* and *encoding* arms, it can be secured into place by preventing the rotation of respective revolute joints to stabilize insertion of the instrument. To this end, a joint brake mechanism is proposed to lock the four proximal joints on the *passive* and *encoding* arms (Figure 2D) across the targeting stages. As shown in Figure 2F, the joint brake mechanism is comprised of a pair of friction rings, which were configured as two anti‐clockwise and clockwise arranged stacked layers, surrounding the installed joint for bidirectional braking effect. To keep the robot compact, small (103 mm × 40 mm) and lightweight (<100 g) tendon‐driven braking actuation units (Figure 2G) are incorporated to drive the brakes via Bowden cable connection,^[^ ^28^, ^29^ ^]^ which keep the actuation units ≈200 mm away from the main robot positioner and head coil (Figure 2A). Rolling‐diaphragm‐sealed hydraulic actuation is introduced to provide a sufficient braking force, even though transmitted through 10‐m long hydraulic pipelines from the control room. This kind of transmission also features high positional frequency response (5 Hz) and small time delay (52 ms).^[^ ^30^ ^]^ However, the everting motion of the rolling diaphragm can only guarantee a limited linear working stroke, which can be further hindered by the transmission lost. A pulley push against the actuation wires is therefore integrated to scale down the working stroke that is required. Details of the joint brake mechanism are described in Section S1 (Supporting Information). To this end, by separating the joint brakes and the actuation units, the main robot attached to the skull remains highly compact with reduced motion inertia from the robot linkages. The 30 mm stroke of the rolling diaphragm can still provide a variable locking force for robot joints, which is favorable in clinical procedures where a damping effect during manual adjustment may be desired.

### 2.2 Narrow Range and Precise Soft Actuation

While the wide‐range instrument positioning is allocated to the surgeon's manual manipulation, we propose a second stage for a precise robotic mechanism that automatically adjusts targeting error to within an acceptable level (e.g., 2–3 mm for DBS). Although the two‐stage adjustment approach sacrifices the ability of full‐range automated positioning, the actuation components can retain a small size due to lower requirements for motion range and output force. The small size importantly allows two independent positioners to be employed simultaneously for bilateral procedures, improving the operation efficiency. The subsequent light weight also enables skull‐mounted usage within the head coil, minimizing effects of undesired patient movement. In terms of fulfilling the strict requirements for MR safety, the mechanism uses soft actuators fabricated from polymers and elastomeric materials, which have garnered increased interest in surgical applications navigated with MRI guidance, e.g., steerable needle interventions,^[^ ^31^ ^]^ as attributed to the inherent compliance and dexterity. In this work, we propose a soft robotic manipulator with a delta mechanism^[^ ^30^ ^]^ architecture for instrument positioning within a localized workspace (±5°). To this end, the two‐stage coarse and fine adjustment provides a total reachable workspace of ±35°, which is sufficient for general stereotactic neurosurgeries.^[^ ^25^ ^]^ More details about the workspace can be referred to Section S2 (Supporting Information). As located distally on the *passive* arm, three soft actuators angled 120° apart push the instrument guide at the central connecting point, as in Figure 2C. The soft actuators are directly connected through 10 m long pipelines to motorized cylinders in the control room. The applied hydraulic actuation approach^[^ ^18^, ^30^ ^]^ paired with FEA‐optimized soft robot design feature low transmission latency (117 ms on average under 1.4 Hz) and hysteresis (0.22°), as well as fine motion resolution (0.058°), which were experimentally validated as in Section S2 (Supporting Information). Relevant results can also be referred to Figure S1A,B (Supporting Information). Unlike conventional rigid delta mechanisms where many passive joints/bearings must work in concert to avoid mechanical interference, the compliant nature of the proposed soft manipulator eliminates this concern, and in fact benefits from the antagonistic forces acting between actuators, as their motions are coupled. The robot performance (e.g., frequency response) benefits due to increased overall stiffness. The degree of this stiffening effect can be even adjusted by the preloading induced in each chamber, which determines their baseline pressure. Additionally, by pooling the efforts of two actuators acting in a similar direction, higher payload capacity can be provided. To maintain the robot RCM during the fine adjustment stage, constant contact between the spherical slider and a corresponding spherical surface (Figure 2C) is enforced because each actuator is angled upwards toward the instrument guide, providing a component of vertical force. Such that the movement of the instrument guide will still be actively followed and encoded, even if the instrument guide is manipulated when the coarse adjustment stage is locked.

A trade‐off of using soft actuators in applications requiring high precision is that their inherent compliance and deformability are still present even with efforts to increase overall stiffness through means such as antagonistic actuation and pressure preloading. As a result, the fine adjustment stage alone remains susceptible to external disturbances. Although both the passive and encoding arms can be locked into place independently by joint brakes (Figure 2A), the final coupling point between the two arms is comprised of the three compliant soft actuators. It effectively separates the arms into two independent serial‐link mechanisms, which lack the structural rigidity of parallel mechanisms that typically requires a closed chain of linkages. To address this concern, granular jamming was implemented to provide a sufficiently strong but reversible shape locking effect for the soft manipulator while maintaining robot compactness. A particle‐filled elastic membrane was anchored on top on the spherical constraint of the passive arm to enclose a portion of the instrument guide (Figure 2D,E), allowing stiffness modulation based on the level of applied vacuum pressure. Details of the shape locking mechanism are described in Section S3 (Supporting Information). Along with the joint locks, the two‐stages locking can provide sufficient overall stiffness (4.07 N mm^−1^) to maintain the targeting trajectory during instrument insertion (please refer to Section S2 and Figure S1C, Supporting Information).

**Figure 3:**
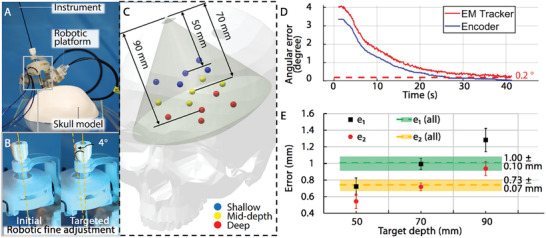
Lab‐based targeting accuracy test of the robotic platform. A) Setup of robot onto a skull model, which is fixed to an acrylic box. A tabletop EM‐field generator was used to acquire instrument tip positions. B) Manipulation of instrument guide from its original position to planned trajectory. C) Virtual targets were selected at three different depths (50, 70, and 90 mm) from the incision point to simulate the real anatomical workspace. D) One of the targeting attempts showing that the soft actuation with a proportional‐integral‐derivative (PID) controller can achieve targeting error as low as 0.2°. E) Results of the final targeting accuracy test using two measurement methods, i.e., error (e_2_) perpendicular to the desired trajectory (radial error) at target depth, as well as error (e_1_) to target (vector error).

A positioning accuracy test on the overall workflow was conducted in the lab environment. Prior to the MRI‐based trials (refer to Section 2.3), the error resulting from mechanical architecture, soft actuator, and controller (refer to section Targeting kinematics under Experimental Section) should be evaluated, without involving error sources under MRI, e.g., marker error, MR image distortion, and robot‐MRI frame registration error. Additionally, extra error induced by manual insertion of the instrument should also be taken into consideration to see whether sufficient transmission stiffness can be provided by the orientational locking mechanism for instrument advancement. The robot was attached on an artificial skull model (**Figure** **3**A) at 65° from the orbitomeatal plane,^[^ ^32^ ^]^ which is commonly referenced for instrument orientation in stereotactic DBS procedures. The electromagnetic (EM) tracking system (Aurora, NDI Medical, Canada) can act as a comparable proxy for the tracking approach under MRI. In this test, the ground‐truth positions of the instrument tip and targets could be obtained by the EM markers, thus realizing registration and accuracy evaluation. Designated points on the robot base were measured to co‐register the robot to the EM tracking system coordinate. A clinical‐grade stainless‐steel stylet and cannula assembly (Ø 2 mm) was adopted as the insertion instrument. Commands were sent to manipulate the instrument guide from its initial position to the desired trajectory (Figure 3B,C). Results showed that the feedback controller could steadily steer the instrument guide to the target orientation with an error of less than 0.2° on average over 10 targets (Figure 3D). A more comprehensive targeting test was also conducted to evaluate the overall targeting accuracy with the proposed workflow (Figure 1D–F), from manual coarse adjustment to instrument insertion. During the test, target depths were chosen to be ≈50, 70, and 90 mm, with five targets each evenly distributed within the workspace, as in Figure 3C. Among them, 90 mm is the typical depth of the region of interest for DBS.^[^ ^33^ ^]^ Once pointing to the target, the instrument guide was locked by both shape locking and joint‐based locking. The phantom cannula assembly was then manually advanced through the instrument guide with a pre‐defined depth. The tip position acquisition was repeated 10 times in every test. There are various approaches for calculating targeting error in stereotactic neurosurgery, including “vector error (e_1_)”, indicating the distance from the center of the effective instrument zone to target, and “radial error (e_2_)”, defined as the distance between the target and the insertion trajectory.^[^ ^1^, ^25^, ^34^, ^35^ ^]^ Among them, radial error (e_2_) is the most cited. As tabulated in Figure 3E, the overall **e_2_** was kept within 0.73 mm on average, and its variation is less than 0.1 mm, demonstrating the system's potential of accurate instrument targeting.

### 2.3 MR‐Based Instrument Tracking and Path Planning with Wireless Markers

Precise localization of robot under MRI is needed to enable accurate positioning of instruments. Development in MR markers via *passive‐*, *active*‐, or *semi‐active* tracking, has enabled precise targeting and control during surgical operations. However, the existing *passive* tracking approaches involve complicated MR sequences to identify marker signals unambiguously from background signals,^[^ ^36^ ^]^ as well as adjacent markers. The use of *active* and *semi‐active* markers is also hindered by either the mandatory long electrical wire connection,^[^ ^37^, ^38^ ^]^ and extra electronic components that increase overall footprint,^[^ ^39^, ^40^ ^]^ thus posing a barrier in clinical or interventional implementations.^[^ ^39^ ^]^ Based on pre‐op 3D imaging, the markers should have high signal‐to‐noise (SNR) for ease of visualization or tracking both visually and with computer algorithms. MR‐based RF markers^[^ ^41^, ^42^ ^]^ that are inductively coupled to the scanner imaging coils have gained increasing interest by offering substantially amplified scanner excitation magnetic field B1 near the marker, while being miniature and wireless. This removes the need for electrically conducting wire connection to the MRI scanner, thus reducing the implementation complexity, as well as the risk of RF‐induced heating due to wiring.^[^ ^37^ ^]^ Furthermore, by using 1D‐projection MRI pulse sequences, such kind of markers can offer real‐time tracking with high SNR at > 30 Hz. However, the limited design approaches of resonant circuits on the markers have also led to orientation dependency^[^ ^41^, ^43^ ^]^ while being visualized under MRI, such that the SNR drops to its minimum when the inductor surface normal aligns to the MRI static magnetic field.

#### 2.3.1 Miniature and Wireless Tracking Markers

**Figure 4:**
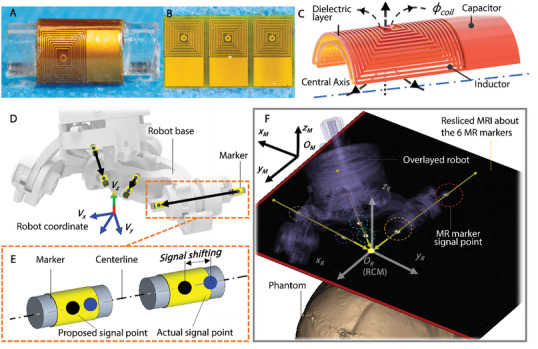
Robot registration in the MRI coordinate with the proposed MR tracking markers. A) Curved form of the resonant circuits forming the omnidirectional marker. B) Original planar form of the resonant circuits. The circuits are fabricated monolithically through FPC manufacturing processes. C) Layout of an individual resonant circuit. The circuit inductively couples to the scanner RF coils in order to amplify the MR signal. D) Three pairs of markers embedded into the robot base with each pair's centerline intersecting at the robot coordinate frame origin. E) Markers are used in pairs to compensate for signal shifting due to assembly error. F) Resliced MR image aligned with the six MR tracking markers. The registered robot pose w.r.t. the target/phantom is shown.

In this work we propose a miniature fiducial marker^[^ ^44^ ^]^ (**Figure** **4**A) to localize the robot base in MRI coordinates. It utilizes a specifically designed monolithic *flexible* printed circuit (FPC) board fabricated with three curved MR resonators. The marker enables wireless 3D positional tracking with strong signal amplification in all orientations. The fabrication process has been simplified by the printing of markers with a monolithic structure (Figure 4B), where the inductor and capacitor are connected to each other (Figure 4C). After printing, the resonant circuits are assembled onto a glass tube (Ø3 × 8 mm) filled with 10 mm Gd‐doped water that acts as an internal signal source. Due to potential assembly error, the imaging signal of the marker may become offset along its geometric long axis, thus shifting the imaging signal under MRI, as shown in Figure 4D. In this case, the marker's imaging signal no longer represents its geometrical center. To eliminate this effect, the markers are employed in pairs, where their long axes are aligned. By this means, a vector unaffected by signal shifting can be formed for each pair of markers by connecting the two markers’ locations revealed by MRI. As in Figure 4D–F, the robot poses w.r.t. the MRI frame can be determined by at least 2 pairs of markers attached to the robot base, forming two vectors pointing to the robot RCM point. By design, the two vectors are placed perpendicularly and aligned with the two joint axes of the robot's base linkage, so that they represent the *x_R_* axis and *y_R_* axis of the robot. The *z_R_* axis can be resolved by the cross‐product of the two vectors. A third, redundant pair of markers is added to improve registration accuracy (Figure 4D). An example robot registration result is shown in Figure 4F, where the six MR tracking markers, robot and phantoms skull can be seen from the resliced images. Using 1D‐projection gradient readouts, the marker positions could even be obtained in real time (>30 Hz), refer to Section S4 (Supporting Information).

#### 2.3.2 MRI Compatibility

**Figure 5:**
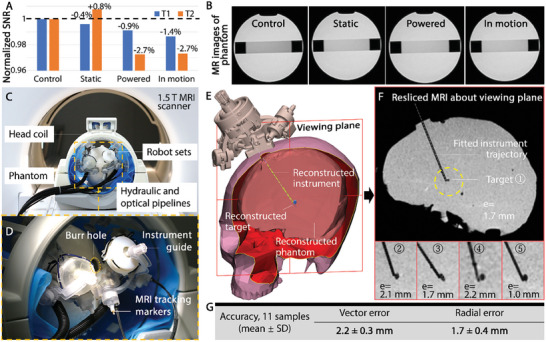
MRI‐based validation of proposed system on phantoms. A) Result of MRI compatibility test. Normalized SNR variation in different robot conditions was displayed. B) MR images of the phantom in the states of control (no robot), static, powered and in motion for calculating the SNR. C) Two robots mounted on a head phantom within a split head imaging coil. D) MRI‐visible markers mounted on the robot base. Burr hole visibility and clearance is maintained, reserving space for the surgeon's observation and operation. E) Virtual robot augmented on the 3D MRI‐reconstructed phantom, mandrel and target. F) Resliced sagittal view along the plane that contains the mandrel trajectory and target simultaneously. Targeting accuracy results are shown for the target samples in five different brain locations. G) MRI‐guided targeting accuracy test result of positioner for stereotactic neurosurgery.

The robotic system is mostly composed of polymer‐based materials, however, trace amounts of conductive metal are implemented in the joint brake mechanism and in the MR‐based tracking marker circuits. Since the proposed robot operates close to the scanner isocenter, imaging artifacts may be induced due to EM interference as a result. To validate MRI‐compatibility, the robot was operated inside a 1.5 T MRI scanner (MR450, GE, USA), with placement close to a commercial MRI phantom (J8931, J.M. Specialty Parts, USA) at the scanner isocenter. The robot and phantom were housed in an imaging head coil (#5 182 594, GE, USA). The T1‐ weighted fast field echo and T2‐weighted turbo spin echo sequences were adopted to obtain the MR images. According to the guidelines by the National Electrical Manufacturer's Association,^[^ ^45^ ^]^ the results of SNR analysis were calculated under the two imaging sequences, as in **Figure** **5**A. Figure 5B shows the resultant MR images of the phantom by T2‐weighted TSE under four different conditions: i) Control: only phantom placed in the scanner; ii) Static: robot involved and remained power OFF; iii) Powered: robot kept still, but with the hydraulic and electric power ON; iv) In motion: robot in operation. The maximum SNR loss in the successive conditions was found to be within 3%, even with the robot in full motion. For reference, ASTM only considers artifacts as image intensity variations of greater than 30%.^[^ ^46^ ^]^ This indicated no observable image artifact was found in the MR images under different robot operation scenarios.

#### 2.3.3 Phantom Trial

To validate the clinical workflow of the proposed system under MRI, a targeting test was performed, as shown in Movie S2 (Supporting Information). A brain phantom fabricated from 4.5% agar gel (Biosharp Inc., China) was chosen as the experiment subject to enhance image contrast for visualization of instrument and target lesions. The experimental setup mimics the real scenario for bilateral stereotaxy. The experimental subject (i.e., skull model with brain phantom) was able to be fitted in the imaging zone of the head coil with the proposed robotic system, refer to Section S5 (Supporting Information). As in Figure 5C, a skull model containing the brain phantom was tightly fixed in a transparent acrylic tube within the head coil, where relative movement between the setup and the head coil was minimized. Two robots were anchored on the skull model at two sides of the sagittal plane independently. Compared to the existing approaches with monolithic robot mounting^[^ ^18^ ^]^ for multiple targets, the proposed independent fixtures allow improved flexibility in setting up robot orientations around the burr hole. Space around the instrument entry (Figure 5D) was also reserved. Multiple ellipsoid‐alike (major × minor axis length = Ø 2 to 4 mm × Ø 4 to 8 mm) silicone beads were imbedded ≈90 mm beneath the skull to mimic the anatomical targets, STN or GPi, for DBS. The targets were shown as negative artifacts under MRI, differentiated from the bright and contrasted signals of the phantom. Six of our custom markers were embedded into the robot base with geometry constraints as in Figure 5D. 3D fast spoiled gradient recalled‐echo (FSPGR) sequences were used to obtain the marker locations and perform robot registration, with parameters: TR/TE = 8/1.8 ms, flip angle = 10°, acquisition matrix = 240 × 240 × 176 (1 mm^3^ voxel size) in sagittal slab.

After which, the computer calculates the desired linear insertion path and depth based on the robot kinematics and the target location acquired. The coarse and fine instrument guide adjustment and the instrument insertion were then performed in one go, during which interactive lighting instruction was active. The above procedure is done by transferring the phantom only one time out of the scanner bore. 3D imaging is repeated for post‐imaging following the manual insertion of a Ø 1.8 mm ceramic mandrel to assess its position relative to the target. To extract the actual mandrel trajectory accurately, 3D reconstruction is conducted based on the negative artifacts presented by the inserted mandrel. The positional relationship between the inserted mandrel, target, and phantom is reconstructed based on MR images in Figure 5E, with the virtual robot overlaid. The linear‐fitted centerline of the 3D‐reconstructed insertion path was used for positional error calculation. The resliced MRI about the viewing plane that revealed the insertion trajectory and the target can be seen in Figure 5F. Note that the silicone targets’ locations may be shifted from their original placement when contacting the mandrel (Figure 5F), as they are relatively “rigid” compared to the gel‐like phantom. This problem should be eliminated in actual cases due to the highly viscoelastic mechanical properties^[^ ^47^ ^]^ of the anatomical targets (e.g., STN and GPi), which are not well differentiated from their surroundings. As tabulated in Figure 5G, with 11 targeting attempts, the overall accuracy (mean ± SD) was recorded as 2.2 ± 0.3 mm (vector error), and 1.7 ± 0.4 mm (radial error), respectively. The decreased targeting accuracy under MRI may be induced by the limited resolution (1.0 mm) and the assembly error of the MR‐based tracking markers for registration. Additionally, using a ceramic mandrel in the phantom may introduce extra error due to the instrument deflection induced by instrument‐phantom interaction when compared to the lab‐based setup.

#### 2.3.4 Cadaver Trial

**Figure 6:**
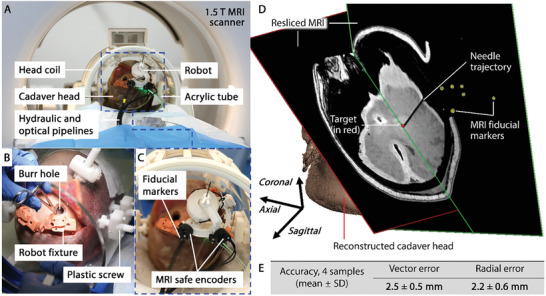
Result of MRI‐based targeting test on cadaver. A) Experimental setup in the 1.5‐T MRI scanner (MR450, GE, USA). B) Cadaver head fixed in acrylic tube using plastic screws. A burr hole was created based on the pre‐op imaging. C) Robot mounted to the skull via robot fixture. D) Post‐op imaging showing one of the targeting attempts. E) MRI‐guided targeting accuracy test result of the cadaver trial.

To validate the proposed robotic platform in human anatomy, we performed a cadaver trial of needle targeting with the proposed robotic system under a 1.5 T MRI scanner (MR450, GE, USA). The experimental setup of cadaver trial is shown in the newly added **Figure** **6**A. A cadaver head was defrosted 48 h before the test and fixed in an acrylic tube mount using plastic screws (Figure 6B). For pre‐op planning, a T1‐weighted imaging model of the brain region was acquired, using 3D FSPGR sequence with inversion recovery preparation. The parameters are: TR/TE = 5.1/2.1 ms, flip angle = 12°, acquisition matrix = 256 × 256 × 392 (1 mm^3^ voxel size) in sagittal slab, preparation time = 450 ms. Based on the pre‐op imaging, a 12.5 mm burr hole was created, where the robot was installed by connecting to a detachable robot fixation (Figure 6C) in between. Such 3D MRI scans were repeated so that co‐registration between the robot and MRI can be performed. Meanwhile, virtual target points were defined by the operator in MRI coordinates, so that coarse and fine instrument guide adjustments and the instrument insertion can be performed in one go for each target points. Post‐op 3D scans were conducted upon each instrument insertion, using the same sequence as registration and pre‐op scanning. The centerline of each needle trajectory (Figure 6D) was fitted for targeting accuracy calculation. As tabulated in Figure 6E with 4 targeting attempts, the overall accuracy (mean ± SD) is recorded as 2.5 ± 0.5 mm (vector error), and 2.2 ± 0.6 mm (radial error), respectively. Unlike the homogeneous Agar phantom in the phantom tests, needle insertion in a more complicated inhomogeneous medium, i.e., defrosted brain tissue, may be the source of extra error. Despite the fact that the MRI measurement resolution is limited to 0.5–1 mm, our cadaveric trial still indicates a satisfactory targeting accuracy to meet our benchmark, which is <3 mm.^[^ ^1^, ^48^ ^]^ Further improvements should be implemented to reduce extra error sources with MRI‐based guidance. For instance, incorporating more fiducial markers to increase the robot‐MRI co‐registration accuracy and increasing the robot's structural rigidity.

## 3 Conclusion and Discussion

This paper presents the design and experimental validation of a robotic positioner for intra‐op MRI‐guided stereotactic neurosurgery. Providing a sufficient workspace (± 35°) in performing instrument targeting, the positioner is lightweight (203 g) and compact (Ø97 mm × 81 mm height), thus enables skull‐mounted usage with up to two robots fits within most standard imaging head coils for unilateral or bilateral procedures. Rather than running in a fully autonomous manner, the system operates in two stages, including wide‐range workspace positioning passed to the surgeon's manual manipulation, followed by soft robotic fine adjustment. Accredited to the integrated optical encoders (0.044° resolution), constant instrument guide orientational tracking can be achieved in addition to the positional feedback from MR images, even without active imaging, e.g., when the subject is transferred out of the scanner. This also makes possible that the instrument targeting, insertion and intervention procedures to be conducted without disruption, smoothening operation workflow for multiple targets under bilateral operation. Due to manual insertion performed by the surgeon, the robot resists external disturbances by both the cable‐driven joint locking mechanism and with granular jamming shape locking, providing a sufficient transmission stiffness of 4.07 N mm^−1^ across the targeting and instrument insertion stage.

The five‐bar linkage structure of the robot provides a natural RCM constraint around the burr hole for targeting while preserving visibility and access for managing operative risks. Precise (<0.2° orientation error) soft robotic instrument positioning is achieved via FEA‐based optimization (see the Experimental Section) on design parameters, including fold numbers, stiffness and diameter of the outer folds, wall thickness, as well as the fluidic preloading value. The optimization balances the soft actuation linearity (R^2^ = 0.9929) within the required manipulation range (<5°), frequency response (1.4 Hz) and soft robot durability, resulting in fine angular motion resolution (0.058°). The system's workflow and targeting accuracy were evaluated under lab‐environment, reporting a radial error <0.8 mm. MRI‐based validations were performed in skull phantoms and a cadaver subject, with radial error <1.7 mm and <2.2 mm, respectively. Custom‐made wireless omni‐directional tracking markers facilitate robot registration under MRI while keeping the system compact. The system generates zero electromagnetic interference, allowing the use of intra‐op MRI guidance during robot actuation and for evaluating the interventional process, which plays an important role in balancing adequate treatment outcomes (e.g., tumor ablation) and tissue function preservation.

With the use of 3D printing for prototype fabrication, system inaccuracies are unavoidably induced through disassembly and plastic deformation of the parallel linkage pivots. Despite this, the robot is able to achieve targeting errors within the clinical design target of <3 mm. For further improved robot performance, it is important to investigate more reliable fabrication approaches, e.g., computer numerical control machining or injection molding. For the proposed applications, there are components that must be suited for intra‐op MRI usage, while at the same time, having adequate mechanical properties. In this case, high‐performance thermoplastic, e.g., PEEK/PEKK or UTEM is a promising alternative to the current prototyping materials. Additionally, medical‐grade properties for components in contact with the patient should be considered, alongside sterilization (e.g., ethylene oxide), despite the fact that the robots are disposable by design. This would lay a foundation for future pre‐clinical validation toward clinical practices.

As a proof‐of‐concept study, the system's potential for intra‐op MRI‐guided stereotactic neurosurgery was demonstrated with a cadaveric trial. Several practical considerations need to be further investigated, such as the setup in MRI, integration with MR scanning coils, and the maintenance of the hydraulic transmission system. A more robust and advanced controller should also be developed to fuse the two sensing modalities, i.e., positional feedback from the optical encoders and real‐time MRI. Despite the installation of many interventional MRI scanners around the world, real‐time MRI remains underutilized and should be promulgated for its powerful intra‐op capabilities.

## 4 Experimental Section

Implementing soft fluid‐driven actuators in accuracy‐demanding applications is still considered challenging due to actuation nonlinearity and variations in fabrication quality. Existing studies^[^ ^49^ ^]^ discovered that the degree of nonlinearity varies along the input‐output spectrum in a soft actuation system. In the below sections, the design optimization toward improved soft robot performances, e.g., actuation linearity and durability were investigated. Besides, a kinematics model was developed for the soft robotic fine instrument adjustment, which was facilitated by a proportional‐integral‐derivative (PID) controller with feedback from embedded optical encoders and MR images.

### Design Optimization for Soft Actuation

**Figure 7:**
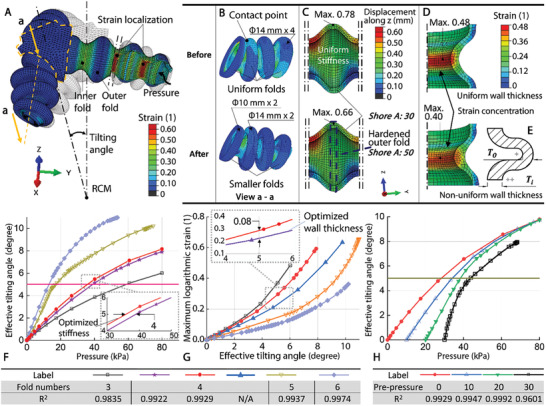
A) FEA of robot end‐effector (orange dashed line) with three actuators tilting around an RCM when having one actuator inflated. B) Comparison of actuators without (top) and with (bottom) optimized fold diameters. C) Comparison of soft chambers without (left) and with (right) optimized stiffness. D) Strain concentration on the inner fold induced by fluidic pressure. Inner fold thickness *T_i_* is 1.2 times of outer fold thickness *T_o_*, reducing strain concentration. E) Cross section schematic indicating the inner fold thickness (*T_i_*) and outer fold thickness (*T_o_*) of the bellow wall. F) FEA result showing the effect of bellow fold number (3 to 6) on actuation linearity within a tilting range of 5°. The linearity between input (pressure) and output (effective tilting angle) is represented by the coefficient of determination R^2^, derived by linear fitting using least‐square regression. G) FEA result showing the effect of bellow fold number (3 to 6) on maximum logarithmic strain. H) FEA result showing the effect of pre‐pressure (0 to 80 kPa) on soft actuation linearity within a tilting range of 5°.

FEA was conducted to understand the complicated robot characteristics while being applied with fluidic pressure, thus facilitating design optimization. To fulfill the workspace requirement (±5°, as in Section 2.2) for the fine adjustment stage while keeping the robot compact, an axisymmetric bellow‐shape design was adopted in the soft actuators that comprise the delta mechanism. Through inflation and deflation, each actuator had a working stroke of 36 mm by elongating 2.8 times from its minimum length of 20 mm. Generally, bellow‐shape soft actuators feature a high stroke‐to‐size ratio and could lengthen by up to 3.4 times.^[^ ^50^ ^]^ In **Figure** **7**A, FEA of the robot end‐effector (orange dashed line) with three actuators tilting around an RCM wass shown when having one actuator inflated. Boundary conditions were applied to the central block so that its movement was constrained on a spherical surface which follows the robot RCM. The robot structure was tessellated primarily with quadratic hexahedron elements (C3D8H), which leads to more efficient computing toward convergence and higher geometric accuracy for (axi‐)symmetric models, as compared to tetrahedrons.^[^ ^51^ ^]^ The elastomers were modelled as isotropic materials and follow a second‐order polynomial hyperelastic approximation. The coefficients related to the specific strain‐stress relationships were obtained through uniaxial testing. During the simulation, a linearly increased‐ramp pressure was applied to the inner surfaces of the selected actuator, which induced the change of the tilting angle of the model. Figure 7B–E shows the contour plots about partially the actuators with and without optimized design parameters, while the effect of design parameters on actuation linearity and maximum logarithmic strain are summarized in Figure 7F–H. Only data within 5° of the effective tilting angle was linearly fitted using least‐square regression, where the linearity between the input (pressure/tilting angle) and output (tilting angle/maximum logarithmic strain) could be presented by coefficient of determination, denoted R^2^. It could be observed that the actuation nonlinearity tends to be localized at the end areas of the soft actuator's working stroke, while the earlier areas present a near‐linear behavior. Therefore, building a linear model was possible by using the working range that was in the near‐linear region.

*Fold Numbers*: Increasing the fold numbers tends to shift the near‐linear regions of the soft actuators within the targeted 5° of effective tilting, with higher fold numbers resulting in greater linearity (R^2^) of the curves in this region, as shown in Figure 7F. Meanwhile, the maximum logarithmic strain of the model decreases with higher fold numbers, as shown in Figure 7F, lowering the material deformation needed to achieve a similar tilting angle. Moreover, the effective tilting angle increases with more folds integrated into the soft actuator (Figure 7G), meaning that less pressure was required to overcome the model rigidity. However, it also indicates that the manipulation stiffness of the delta‐mechanism decreases with more bellow folds, such that a lower output force was generated to push against the loading. Enhancing the model stiffness could improve the soft manipulator's hysteresis and responsiveness, benefiting the instrument positioning. However, this would result in high actuation pressures which could cause higher local stresses and subsequent rupturing. Making use of the high linearity at the near‐linear region of the soft actuation could also ease the demand of building a sophisticated controller. Based on the results, four was the optimized numbers of folds, balancing the trade‐offs between manipulation stiffness, soft actuation linearity and potential for local material rapture.

*Stiffness and Diameter of the Outer Folds*: Generally, chambers made purely from elastomeric materials could not withstand high pressures, limiting their loading capabilities and may result in uncontrolled expansion upon pressurization. Previous studies^[^ ^12^ ^]^ proposed to restrict the radial expansion of soft chambers with fiber braided sheath or spring reinforcement, etc., which constrained their deformation to 1D elongation. However, such reinforcement surrounding the soft chambers also changes the robot's architectural composition, hindering its material compliance, and reducing the adaptability to environmental or mechanical constraints. This prohibits applications where the chambers act as both actuators and joints, which demands a certain level shape adaptation capability. To this end, an actuator design with non‐uniform stiffness is proposed, as in Figure 7C. To restrict the undesired radial expansion while not hindering the flexibility of the actuator, a small portion (2 mm in width) around the peak of the outer folds was hardened (FLX9050, Shore A hardness 50) by mixed 3D printing with rigid photopolymer. The stiffness difference shifts the deformation toward elongation while the soft chamber was being applied with fluidic pressure. This could also lead to improved actuation linearity (Figure 7F) within a small range (0–5°) of manipulation. The remaining parts of the soft actuator was 3D printed from elastomeric materials, i.e., Agilus (Shore A hardness 30), which comprises the bellow itself, with rigid materials (VeroWhitePlus) integrated as flanges at both ends for connecting to adjacent structures and to the hydraulic transmission. Although 3D printing was chosen as a preliminary fabrication approach for the benefit of rapid prototyping, the presented robotic system can also be made from molded medical‐grade plastics and elastomers. As shown in Figure 7B, to reduce the blocking of movements between the adjacent folds, a smaller diameter (10 mm) was applied to the outer folds at the two ends of the actuator, while the remaining outer folds in between were kept at 14 mm, which was constrained by the size of the robot. With this approach, the ability of the actuators to act as passive joints could be guaranteed.

*Wall Thickness and Fluidic Preloading*: A trade‐off of using 3D printing in the current stage was that the longevity of components was generally lower due to repeated strain and material imperfections, which could create local peaks in strain that cause formation of microscopic cracks and eventually result in fatigue failure.^[^ ^52^, ^53^ ^]^ This raises the concern related to the strain/stress concentration on the inner folds of the bellows while being applied with fluidic pressure, which could be observed from the FEA contour plot (Figure 7A,D). To further reduce the possibility of local material rapture and improve durability, a non‐uniform wall thickness design (Figure 7E) was adopted. Such a method was reported^[^ ^54^ ^]^ to differentiate the property of the inner and outer folds in a bellow structure to reduce the stress/strain localization along the actuator length. In this case, the wall thickness ratio on the inner and outer folds was found to be 1.2. In a soft fluidic‐driven actuation system, preloading could reduce the backlash and enhance the transmission efficiency. The FEA results (Figure 7H) also show that the actuation linearity (R^2^) improves with higher preloading until it reaches 20 kPa.

### Targeting Kinematics

**Figure 8:**
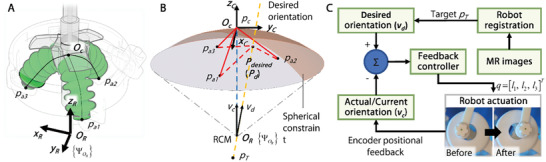
Kinematics representation of the soft robot with three soft fluidic‐driven actuators. A) Schematic diagram showing three actuators pushing against the initial center point *O_c_* in the robot frame $\left\{\Psi_{O_{R}}\right\}$. B) Calculation of the current instrument guide orientation *v_c_*, which can be denoted by the current chamber lengths, i.e., the Euclidean distances between current center point *p_c_* and the chamber base points *p_ai_* (*i* = 1, 2, 3). C) Feedback control loop for targeting. The encoders track the angular positions of the instrument guide, allowing feedback control of the soft actuator.

The five‐bar linkage's kinematics was explored and discussed in the existing research works.^[^ ^27^ ^]^ To achieve automatic instrument orientational positioning in the fine adjustment stage, a kinematics model was built for soft robot actuation on top on the five‐bar linkage's kinematics. The schematic diagram of the soft actuator is depicted in **Figure** **8**A,B. After the coarse adjustment of the robot, the actuator status was locked, where the chambers were at rest as the actuator's initial status. The robot coordinate frame $\left\{\Psi_{O_{R}}\right\}$ was defined at the RCM point *O_R_* for fine adjustment. The instrument guide could be manipulated around the RCM. The task space was defined as the 3D position of the instrument tip *w.r.t*. the robot frame. The initial orientation of the instrument guide was along the *z*‐axis of the frame, and the actuation block was at the point *O_C_* of the spherical surface. The base points of the three chambers were denoted by *p*_*a*1_, *p*_*a*2_, and *p*_*a*3_. Registration between MRI coordinate system {Ψ_*M*_} and robot frame $\left\{\Psi_{O_{R}}\right\}$ was performed first, such that the coordinate of a given target point *p_T_ w.r.t*. $\left\{\Psi_{O_{R}}\right\}$ could be obtained for fine adjustment.

With actuation inputs from the soft chambers, i.e., *q* = [*l*_1_,*l*_2_,*l*_3_]^*T*^, the actuation block could be actuated to the new position *p_c_*. Note that its motion was within the spherical surface constrained by the spherical slider. The coordinate of *p_c_ w.r.t*. $\left\{\Psi_{O_{R}}\right\}$could be calculated by:

(1) $$l_{i}=\left\|p_{c}-p_{ai}\right\|,i=1,2,3$$

The orientation of the instrument guide *v_c_* can be denoted by:

(2) $$v_{c}=\frac{p_{c}-O_{R}}{\left\|p_{c}-O_{R}\right\|}$$

Given a insertion depth *d_i_* (*i* = 1, 2, 3), defined from *p_c_* to the instrument tip *p_tip_*, the position of the tip *w.r.t*. $\left\{\Psi_{O_{R}}\right\}$ can be obtained by:

(3) $$p_{tip}=p_{c}-d_{i}\cdot v_{c}$$

The desired orientation of the instrument guide, *v_d_ w.r.t*. $\left\{\Psi_{O_{R}}\right\}$, can be denoted as:

(4) $$v_{d}=\frac{O_{R}-p_{T}}{\left\|O_{R}-p_{T}\right\|}$$

To solve the inverse kinematics with the target instrument tip point *p_T_*, the desired insertion depth and the desired position of the actuation block can be obtained by solving the equation set of (2), (3), and (4), with the conditions that *p_c_* is located simultaneously in the direction of *v_d_* and on the spherical surface. In the end, the desired inputs of the three chambers can be solved by substituting *p_c_* into (1). The desired encoder angles can also be calculated based on the desired orientation *v_d_*. Based on the above targeting kinematics, the instrument orientation can be adjusted until the detected actual orientation *v_c_* aligns with the desired orientation *v_d_*. Two MR‐safe optical absolute rotary encoders (MR431, Micronor Inc., USA) with a resolution of 0.044° were employed, so as to provide the angular position of the instrument guide to the controller. They are placed on the two proximal joints of the encoding arm (Figure 2B,D). As shown in Figure 8C, once the coarse adjustment was locked, the soft actuator was controlled by a PID controller to steer the needle guide toward the target orientation. The two absolute optical encoders placed on the proximal joints of the encoding arm (Figure 2B,D) could feed back the corresponding angular positions in real‐time. Based on the targeting kinematics, the needle guide orientation could be obtained with the positional information offered by the encoders, thus closing the control loop for needle guide manipulation. In the end, the three soft actuators could be precisely adjusted individually by such a closed‐loop control approach, thus steadily manipulating the needle guide to the target orientation.

## Conflict of Interest

The authors declare no conflict of interest.

## Author Contributions

K.W.K. performed conceptualization, funding acquisition, and project administration. Z.G., H.C.C., D.T.M.C., W.S.P., I.I., R.H.T., and K.W.K. performed supervision.W.S.P., D.T.M.C., and K.W.K. performed investigation. Z.H., G.F., J.D., H.S.T., C.L.C., Z.G., and K.W.K. performed methodology. Z.H., L.L., J.D., and H.S.T. performed Experiment/Validation. Z.H., J.D., X.W., and K.W.K. performed visualization. Z.H., J.D., and H.S.T. performed software. Z.H., J.D.L.J., J.D., X.W., and K.W.K. performed wrote the original draft. All authors wrote, review, and edited the draft.

## Supporting information

Supporting Information

Supplemental Movie 1

Supplemental Movie 2

## Data Availability

The data that support the findings of this study are available from the corresponding author upon reasonable request.
