# Supplementary material for: Interactive Multi‐Stage Robotic Positioner for Intra‐Operative MRI‐Guided Stereotactic Neurosurgery
Source: Adv Sci (Weinh). 2023 Dec 10;11(7):2305495. doi: 10.1002/advs.202305495 (PMC10870025; doi:10.1002/advs.202305495)
Supplement: Supplementary file 1 — Supporting Information [file ADVS-11-2305495-s003.pdf]

ADVANCED SCIENCE
Open Access

Supporting Information

for *Adv. Sci.*, DOI 10.1002/advs.202305495

Interactive Multi-Stage Robotic Positioner for Intra-Operative MRI-Guided Stereotactic Neurosurgery

Zhuoliang He, Jing Dai, Justin Di-Lang Ho, Hon-Sing Tong, Xiaomei Wang, Ge Fang, Liyuan Liang, Chim-Lee Cheung, Ziyan Guo, Hing-Chiu Chang, Iulian Iordachita, Russell H. Taylor, Wai-Sang Poon, Danny Tat-Ming Chan and Ka-Wai Kwok**

Supporting Information

Title

Interactive Multi-stage Robotic Positioner for Intra-operative MRI-guided Stereotactic Neurosurgery

Authors

Zhuoliang He, Jing Dai, Justin Di-Lang Ho, Hon-Sing Tong, Xiaomei Wang, Ge Fang, Liyuan Liang, Chim-Lee Cheung, Ziyan Guo, Hing-Chiu Chang, Iulian Iordachita, Russell H. Taylor, Wai-Sang Poon, Danny Tat-Ming Chan and Ka-Wai Kwok*

*Correspondence to: kwokkw@hku.hk

This PDF file includes:

Supporting Discussion S1: Details of joint brake mechanism

Supporting Discussion S2: Experimental evaluation of robot performances

Supporting Discussion S3: Details of granular jamming shape locking mechanism

Supporting Discussion S4: 1D-projection imaging for the proposed MRI markers

Supporting Discussion S5: Desired system arrangement in MRI head coil

Fig. S1: Results of hysteresis, frequency response and transmission stiffness tests

Fig. S2 Result of 1D-projection imaging test for the proposed MRI markers

Fig. S3 Diagrams showing the desired system arrangement in an MRI head coil

Other supporting information for this manuscript include the following:

Movie S1 (.mp4 format): Overview of the robotic positioner for intra-operative MRI-guided stereotactic neurosurgery

Movie S2 (.mp4 format): MRI-based targeting accuracy validation

Supporting Discussion S1

As shown in **Figure 2F**, to execute the braking effect, the friction ring (inner/outer) is one end fixed on the installed joint while the other end is connected to a tendon which pulls the ring downwards into contact with the joint when being actuated. The braking effect is further guaranteed by incorporating rubber sheath with high coefficient of friction. In the Bowden cable transmission, thin (Ø 0.2 mm) tendons (nickel titanium, Ni-Ti) were tightly channeled through PEEK overtubes (OD × ID = Ø2 mm ×Ø0.5 mm). The sheath is axially stiff enough to ensure effective power transmission while preventing the patient skull from being damaged by pulling force. Flexibility of the tendon transmission is still maintained even under high tensile loading. Wire attachment location (**Fig. 2G**) on the actuation unit is also adjustable to reduce mechanical backlash with adequate level of wire pre-tensioning.

As proposed, to keep the main robot on the patient skull compact and lightweight, tendon-driven mechanism was adopted paired with Bowden cable transmission, which is driven by hydraulic transmission connected to the control room. Typically, such a hybrid hydraulic and tendon actuation method would require constant hydraulic pressure on corresponding tendons for constant locking. Since the main goal is simply to keep the robot linkages in place, a retractable mechanism was introduced as a binary switch (**Fig. 2G**), which can be engaged and disengaged while being applied with hydraulic pressure. High strength PEEK springs were utilized to reset the retractable mechanism in countering the hydraulic residual forces.

Supporting Discussion S2

Workspace

While the arc angles of each linkage in the *passive* and *encoding* arms were mirrored, their values determine the workspace of the instrument guide. Generally, the larger the arc angle, the greater the workspace, however, this also results in a bulkier system. In seeking a balance between robot size and workspace, the arc angles of the proximal and distal linkages were chosen to be 70° and 60°, respectively, resulting in a workspace of ±38° coverable by coarse adjustment. To avoid working on the workspace boundary, mechanical constraints applied on the revolute joints limit its actual covered workspace to ±30°. With the inclusion of the final fine adjustment stage, the total workspace is expanded with an additional 5°, providing a total reachable workspace of ±35°, which is sufficient for general stereotactic neurosurgeries *(25)*.

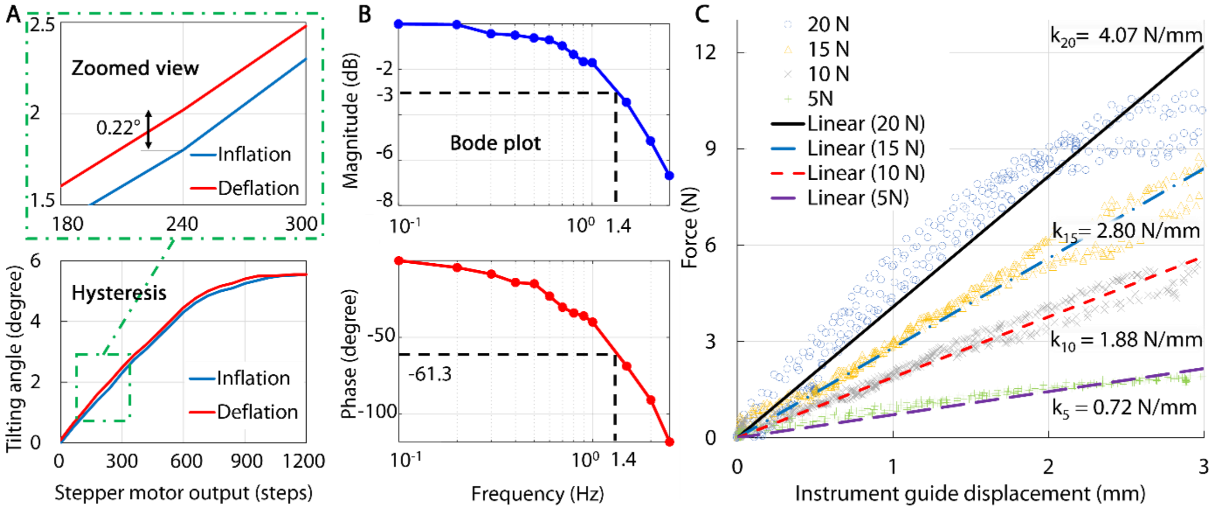

Fig. S1 Result of soft robot hysteresis, frequency response and overall robot transmission stiffness tests. (A) Result of hysteresis test on the soft actuator during inflation and deflation. **(B)** Result of frequency test on the soft actuation. The input of the frequency response test was positional signal to an electric motor, and the output was displacement of the soft actuator. The bandwidth was 1.4 Hz at the cut-off frequency (-3 dB magnitude). The phase lag was kept under 62°. **(C)** Force-displacement plot of the stiffness test results, including results from both loading directions. The actuation wire driving the joint brake was tensioned at four levels (5, 10, 15 and 20 N). Least-square regression is adopted to fit the experiment data linearly, as the robot stiffness is represented by the slope of the fitted lines. In this case, the robot stiffness increased from 0.72 N/mm to 4.07 N/mm, when wire tension was increased from 5 N to 20 N.

Motion resolution and hysteresis

Motion resolution specifies the smallest increment that a linear system can measure or display. To this end, the actuation linearity of the soft robot is experimentally validated with the result of angular motion resolution of 0.058°, slightly smaller than the inherent resolution of the embedded optical encoders (0.044°). At the insertion depth for general DBS procedures, i.e., 90 mm, it represented 0.63 mm of the vector error, which demonstrated the system's great

potential to achieve sufficient positioning accuracy (< 2-3 mm) with a simple feedback controller. Hysteresis of the soft actuator during inflation and deflation was also tested. The motor output, i.e., displacement, which is represented by the motor steps, and the corresponding tilting angle of the instrument guide were recorded. **Figure S1B** shows that the soft robot has a maximum hysteresis of 0.22°, which showed no significant impact on robot control. This is attributed to the stiffened outer fold design of the soft actuator and the hydraulic pre-loading, which improve the overall stiffness.

Frequency response

The dynamic performance, i.e., frequency response of the bellow-shape soft robot with hydraulic actuation was tested. For comparison, the tests were also conducted to a prototype without optimization of wall thickness and stiffness. Bode plot (**Fig. S1C**) showing the dynamic response of the soft actuators was generated by Fourier analysis. The bandwidths are approximately 1.1 Hz and 1.4 Hz, for actuators before and after optimization, respectively, corresponding to their cut-off frequency of -3 dB magnitude. This indicates that the positional command should be updated/changed with the frequency under 1.1Hz and 1.4 Hz, for actuators before and after optimization, respectively, otherwise it could be filtered by the soft robotic system. It also indicates that the FEM optimization for the balance on structural rigidity, soft manipulator durability and actuation linearity also improves the bandwidth of the soft manipulator. The bandwidth was intrinsically governed by the material properties, e.g., stiffness, elastic/ Young's module, which could be improved by fabricating the soft chambers in a stiffer manner. Besides, for the optimized prototype, the phase lag is kept less than 62° within the bandwidth. The transmission latency, or time delay from the input to output, is measured as 117 ms on average.

Transmission stiffness

During the insertion of instruments, tissue inhomogeneity, tissue deformation, and thus instrument deflection can exert as external disturbances to the guiding device resulting in instrument misplacement. To guarantee a stable instrument steering during the insertion, robot's ability to resist external disturbances, i.e., transmissional stiffness is evaluated. Four movable linkages were locked into place by joint braking, with the Bowden-cable transmission pre-tensioned at 5, 10, 15 and 20 N to evaluate the transmission stiffness as it varies with the amount of wire tension. The granular jamming was also taking effect by applying vacuum, securing the soft actuators, which were preloaded by the hydraulic transmission. Such that the instrument guide was appropriately constrained. Besides the robot, a high-precision sliding platform was also installed on the test bed, which can be advanced horizontally to cause a displacement of the instrument guide**.** A high-precision force sensor (Nano17, ATI) with 5-Nm sensitivity was used to measure the axial force generated. The displacement at the contact point was measured by a 6-DoF EM tracking coil (Aurora, NDI). The advancement of the sliding platform was repeated 10 times for each test. **Figure. S1A** illustrates the force-displacement plots of the assembled and fully locked robot. Least-square regression method is used to linear fit the acquired data. The interaction force between brain tissue and instruments is typically under 0.8 N. Compared to the stiffness of the fully locked robot, which is 4.07 N/mm, such interaction force can only cause 0.20 mm displacement to the instrument tip. The experiment result demonstrates the potential of the robot system to maintain the pose of instrument while interacting with tissue, e.g., instrument insertion.

Soft actuator durability

The soft chamber reliability (e.g., related to material fatigue) should align with a desired product lifespan, even with the intention to be a single-use device. The following tests were conducted to evaluate the fatigue life of the soft actuator: ***1)*** Cycle inflation and deflation of the soft

chambers to achieve a 5° of tilting towards a single direction; ***2)*** Trajectory "O" following covering the boundary of ±5° workspace; Pressure holding to maintain a 5° of tilting angle with ***3)*** one chamber inflation and ***4)*** two chamber inflation. The above three tests represent the extreme scenarios that the soft actuators may encounter during application as an instrument guide manipulator. Results show that the actuators reached 5,112.40 ± 558.04 cycles and 1,387.29 ± 132.12 cycles, respectively for task ***1)*** and ***2)***, before their membrane suffered a puncture causing fluidic leakage. The robot is also able to finish tasks ***3)*** and ***4)*** with run time 2.51 ± 0.14 hours and 2.29 ± 0.17 hours, respectively. This demonstrates the potential of the soft actuators to be used in the MRI-guided bilateral stereotactic neurosurgery (e.g., deep brain stimulation, DBS), which generally takes 2 to 3 hours for the whole procedure, even not accounting for the improved efficiency with the proposed system.

Supporting Discussion S3

When the elastic membrane is inflated or kept at atmospheric pressure, it is malleable and can be passively manipulated, i.e., by the pushing force from the soft actuator. When air is evacuated from the sealed elastic membrane, the particles compress together, creating friction between particles to lock the instrument guide at the given orientation *(ref1)*. Studies have investigated the effect of different granule sizes, shapes, and surface finish on overall locking performance *(ref1, ref2)*. For example, granules with fewer faces have been shown to provide enhanced locking, however, at the expense of increased friction when in the non-locked state. To this end, 2 mm diameter PVC spheres with smooth surfaces are adopted to not significantly hinder the instrument guide movement while still providing sufficient stiffness at the locked state.

Supporting Discussion S4

One-dimensional (1D) gradient readouts with non-spatially-selective RF excitation *(ref3, ref4)* were employed to retrieve MR projection signal of the markers, with parameters: TR/ TE = 8.70/2.04 ms, Flip Angle =3°, FOV = 240 mm, resolution = 1 mm/pixel. Dephasing gradients were also applied in the other two orthogonal directions to dephase signals with a spatial periodicity of 10 mm and make the miniature markers more visible *(ref5)*. **Fig. S2A** shows the 1D projected signal intensity of six markers along 3 principal axes under MRI, i.e., x_M, y_R and z_R. Note that the high SNR of the markers allowed them to be contrasted and observed at each projection axis, even within the full field of view, i.e., 0 - 240 mm, including the signals from brain phantom, actuation liquid, etc., as background noise. While the peaks of markers can be easily extracted, their localization can be achieved by the sub-pixel localization method *(ref6)*, in which the marker positions were calculated with intensity linear interpolation (ILI). Note that the lowest SNR of the markers, as can be seen in **Fig. S2A,** was recorded as around 3. For reference, localization by ILI method can take effect as long as the SNR is greater than the signal variance, which is generally less than 2. It can be observed in **Fig. S2A** that signal peaks of each marker in the one projection axes are not consistent magnitudes. This phenomena could be induced by 1) manufacturing error causing a variable quality factor, which is the deviation of the resonant frequency from the ideal (Larmor) frequency, 63.87MHz, for a 1.5T scanner; 2) Orientation dependency still exists, though the markers can still be visualized in all projection axes; 3) Imperfect sensitivity profile compensation of the imaging coil, where the signal intensity of the imaged object is dependent on its distance to imaging coil.

For high-frequency (>30 Hz) real-time tracking, the 3D positions of markers can be resolved by known geometry constraints, e.g., the relative position of markers measured against the possible combination of 1D marker coordinates, even when several of the markers are overlapped. Another approach to avoid the ambiguity induced by maker signal overlapping is to create a linear gradient for slicing in an arbitrary axis by tuning of gradient coil combinations.

The result is such that x_M, y_R and z_R are no longer corresponded to the axial, coronal and sagittal directions of the scanner.

Fig. S2B shows the markers' signal-to-noise ratios from a repeated acquisition experiment (TR/TE = 8.70/2.04 ms, flip angle = 1°, resolution = 1 mm/pixel, FOV = 240 mm). Signal-to-ground ratios are similar and not shown here. In this experiment, three markers were attached to one subject's head, and 1000 repeated 1D-projection signals in three orthogonal directions were acquired when the subject kept still. Signal-to-noise ratios of one marker's data from the 1st, 50th, 100th, 150th projection signals obtained in the three orthogonal (LR, SI, AP) are shown. Note that the fast-tracking pulse sequence we used for 1D-projection signals is modified based on the SPGR sequence, and in our current setting the eight 1D-projection signals acquired in the first eight TRs would be dropped automatically by scanner. We assume the steady state would be reached during 8 TRs. Therefore, the first 1D-projection signal we obtained was acquired in the 9th TR. According to the data, we believe markers had steady signal intensity after eight TRs, and all data we obtained should come from steady state. In the future we could also use 1D-projection signals from the first eight TRs for tracking, since markers' signals before steady state should be larger than their signals in the following TRs and are also sufficient for tracking use.

A

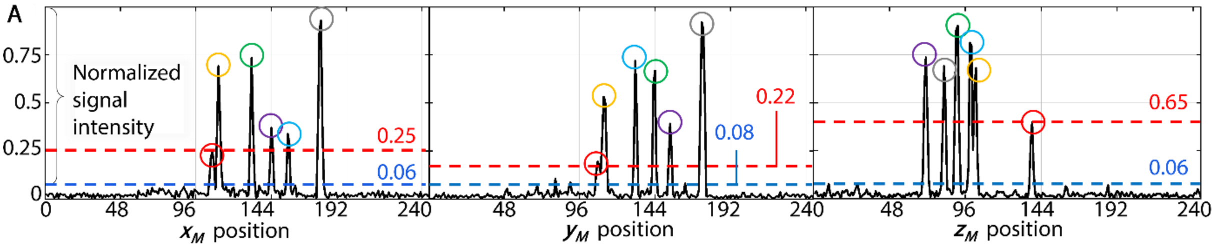

B

Projection direction \ No. of signal obtained		1st	50th	100th	150th
Signal-to-noise Ratio (SNR)	AP	7.168	6.530	6.905	7.014
	LR	6.276	6.669	6.412	6.880
	SI	5.974	5.905	5.985	6.073

Fig. S2 Result of 1D-projection imaging test for the proposed MRI markers. (A) 1D projected signal intensity of six markers along the three principal axes under MRI. The red and blue dotted lines indicate the highest signal peak of the background noises and the lowest signal peak of the six markers. **(B)** Result of signal-to-noise test with repeated 1D projection acquisition.

Supporting Discussion S5

The split head coil/birdcage head coil was used to receive signals for marker tracking and phantom or head imaging. The desired system arrangement in the imaging space (Ø300 × 360 mm) of the head coil (#5182594, GE, USA) is shown in **Fig. S3**, and was employed in the MRI-based tests of this study. The experimental subject (i.e., skull model with brain phantom, or cadaver head) could fit in the head coil with two independent robots installed, accredited to the robot's compact (Ø97 × 81 mm) and lightweight (203 g) design. While **Fig. S3A** shows the actual physical dimension of the selected head coil, **Fig. S3B** shows the coronal view when the system fits in the head coil. The dashed line indicates the workspace where the coil provides sufficient signal sensitivity. The workspace was calculated from one coil sensitivity measurement experiment with a homogenous phantom.

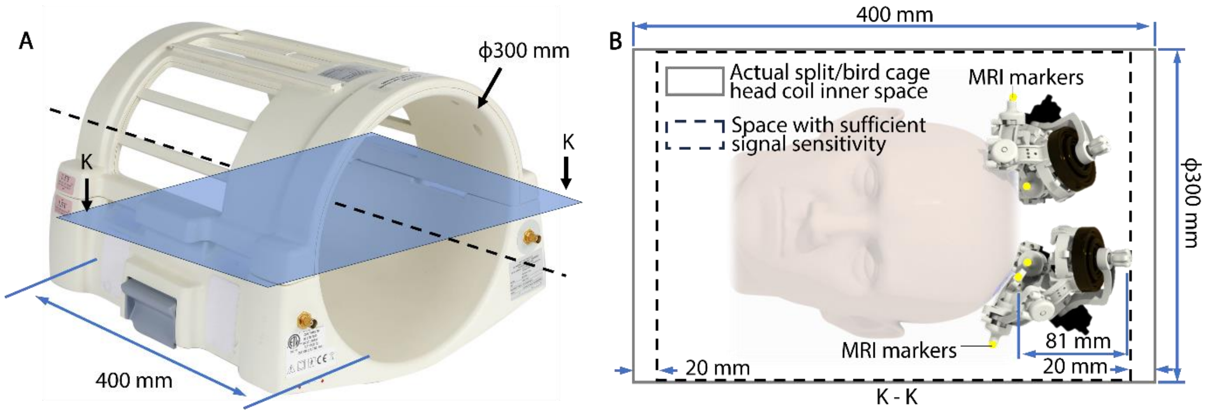

Fig. S3 Diagrams showing the desired system arrangement in an MRI head coil. (A) Physical dimensions of the selected head coil #5182594 (GE, USA). **(B)** Coronal view K-K with the system placed inside the head coil.

Additional References

ref1. M. Cianchetti, T. Ranzani, G. Gerboni, T. Nanayakkara, K. Althoefer, P. Dasgupta, A. Menciassi, Soft robotics technologies to address shortcomings in today's minimally invasive surgery: the STIFF-FLOP approach. *Soft Rob.* **1**, 122-131 (2014).

ref2. A. Jiang, A. Ataollahi, K. Althoefer, P. Dasgupta, T. Nanayakkara, A variable stiffness joint by granular jamming, in *Proceedings of the International Design Engineering Technical Conferences and Computers and Information in Engineering Conference,* 2012, vol. 45035, pp. 267-275.

ref3. S. Weiss, T. Schaeffter, K. Luedeke, C. Leussler, D. Holz, K. Nehrke, V. Rasche, R. Sinkus, Catheter localization using a resonant fiducial marker during interactive MR fluoroscopy, in *Proceedings of the ISMRM Scientific Meeting and Exhibition,* 1999, pp. 1954.

ref4. C. L. Dumoulin, R. P. Mallozzi, R. D. Darrow, E. Schmidt, Phase ‐ field dithering for active catheter tracking. *Magn. Reson. Med.* **63**, 1398-1403 (2010).

ref5. M. E. Tiryaki, M. Sitti, Magnetic Resonance Imaging ‐ Based Tracking and Navigation of Submillimeter ‐ Scale Wireless Magnetic Robots. *Adv. Intell. Syst* **4**, 2100178 (2022).

ref6. M. Rea, D. McRobbie, H. Elhawary, Z. T. Tse, M. Lamperth, I. Young, Sub-pixel localisation of passive micro-coil fiducial markers in interventional MRI. *Magn. Reson. Mater. Phys., Biol. Med.* **22**, 71-76 (2009).
